# Neo-CheckRay: radiation therapy and adenosine pathway blockade to increase benefit of immuno-chemotherapy in early stage luminal B breast cancer, a randomized phase II trial

**DOI:** 10.1186/s12885-021-08601-1

**Published:** 2021-08-06

**Authors:** Alex De Caluwé, Laurence Buisseret, Philip Poortmans, Dirk Van Gestel, Roberto Salgado, Christos Sotiriou, Denis Larsimont, Marianne Paesmans, Ligia Craciun, Drisis Stylianos, Christophe Vandekerckhove, Fabien Reyal, Veys Isabelle, Daniel Eiger, Martine Piccart, Emanuela Romano, Michail Ignatiadis

**Affiliations:** 1 Institut Jules Bordet, Université Libre de Bruxelles, Rue Héger Bordet 1, 1000 Brussels, Belgium; 2 Iridium Kankernetwerk & University Antwerpen, Antwerp, Belgium; 3 GZA, Antwerp, Belgium; 4 Institut Curie, Paris, France

**Keywords:** Early luminal B breast cancer, Priming, Stereotactic body radiation therapy, Anti-CD73, Anti-PD-L1, Neo-adjuvant chemotherapy

## Abstract

**Background:**

Residual breast cancer after neo-adjuvant chemotherapy (NACT) predicts disease outcome and is a surrogate for survival in aggressive breast cancer (BC) subtypes. Pathological complete response (pCR) rate, however, is lower for luminal B BC in comparison to the triple negative (TNBC) and HER2+ subtypes. The addition of immune checkpoint blockade (ICB) to NACT has the potential to increase pCR rate but is hampered by the lower immunogenicity of luminal B BC. Novel strategies are needed to stimulate the immune response and increase the response rate to ICB in luminal B BC.

**Methods:**

The Neo-CheckRay trial is a randomized phase II trial investigating the impact of stereotactic body radiation therapy (SBRT) to the primary breast tumor in combination with an anti-CD73 (oleclumab) to increase response to anti PD-L1 (durvalumab) and NACT. The trial is designed as a three-arm study: NACT + SBRT +/− durvalumab +/− oleclumab. The result at surgery will be evaluated using the residual cancer burden (RCB) index as the primary endpoint. Six patients will be included in a safety run-in, followed by a randomized phase II trial that will include 136 evaluable patients in 3 arms. Inclusion is limited to luminal B breast cancers that are MammaPrint genomic high risk.

**Discussion:**

combination of ICB with chemotherapy in luminal B BC might benefit from immune priming agents to increase the response rate. As none have been identified so far, this phase II trial will evaluate SBRT and oleclumab as potential immune priming candidates.

**Trial registration:**

trial registered on ClinicalTrials.gov (NCT03875573) on March 14th, 2019.

## Background

Luminal breast cancer (BC) is characterized by a positive estrogen receptor (ER) status and categorized into two subclasses, A and B [1]. Luminal B BC has a higher tumor cell proliferation rate than luminal A BC, with a breast cancer-specific mortality rate twice as high [1]. The rates of pathological complete response (pCR) with neo-adjuvant chemotherapy (NACT), are poor for luminal B compared to the non-luminal subtypes: 15% in luminal B versus 46% in HER2 positive BC and 45% in triple negative BC (TNBC) [2–4]. Luminal B BC patients who don not achieve pCR after NACT have a significantly lower event-free survival, revealing a need to increase response rate to NACT for luminal B BC [5].

Recent developments in immuno-oncology permit classification of tumours according to their immunological contexture: inflamed cancer types, such as melanoma and lung cancer, are characterized by the presence of tumour infiltrating lymphocytes (TILs), high CD8+ T-cell density, and high programmed cell death receptor ligand 1 (PD-L1) positivity of tumour or immune cells, and immune signatures. Because of this, higher overall long-term outcomes with immunotherapy can be attained in inflamed cancer types [6, 7], whereas in non-inflamed cancer types, such as a large proportion of luminal B BC, results with isolated PD-1/PD-L1 blockade are disappointing. Hence, strategies to prime immune responses seem critical to increase clinical benefit of immunotherapy in non-inflamed cancer types [8–10]. An area of active research is how to convert non-inflamed cancers into inflamed cancers, leveraging the effects of local and/or systemic treatment and increasing pCR rates with immunotherapy combinations.

The priming of an anti-tumour immune response in early luminal B BC could be attained via a myriad of strategies [11]. In the Neo-CheckRay trial, three priming strategies are used: 1. chemotherapy (given in the three study arms), 2. radiation therapy to the primary BC (given in the three study arms) and 3. blockade of the adenosine pathway (only given in arm 3). The rationale behind these priming strategies will be addressed in the discussion section of the present manuscript.

## Methods/design

### Study design

**Fig. 1:**
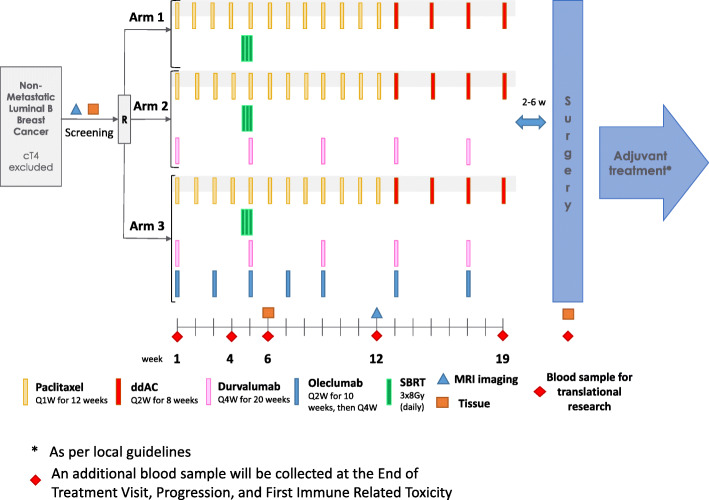
Study treatments of the phase II randomised trial. The safety run-in is equivalent to arm 3 of the phase II randomised trial

The Neo-CheckRay clinical trial is a multicenter, open-label phase II study that randomizes patients with early stage luminal B BC who are candidate for NACT in a 1:1:1 ratio in 3 arms (Fig. 1):

1. Arm 1: the combination of weekly paclitaxel followed by dose-dense doxorubicin-cyclophosphamide (ddAC) and pre-operative radiation therapy (boost dose) on the primary tumour.
2. Arm 2: treatment of arm 1 with the addition of the anti-PD-L1 antibody durvalumab
3. Arm 3: treatment of arm 2 with the addition of the anti-CD73 antibody oleclumab

A safety run-in with inclusion of six patients is planned before starting the randomized phase II part. Those six patients will receive the combination of chemotherapy with durvalumab, oleclumab and SBRT (at the same doses of the IMPs (investigational medicinal product) at identical individual patient timelines than study treatment arm 3).

### Objectives and endpoints

#### Safety run-in objective and endpoints

The objective of the safety run-in is to evaluate the safety and toxicity to perform SBRT (stereotactic body radiation therapy) directed to the primary tumour in combination with durvalumab-oleclumab-paclitaxel and to evaluate the feasibility of performing adequate surgery (breast conserving surgery or mastectomy) without treatment-induced delays. Evaluation of these objectives will be performed by measuring the following endpoints: 1) occurrence of immune related or radiation therapy related toxicity; 2) feasibility of delivering 80% of the total planned dose of paclitaxel and ddAC; 3) feasibility of performing surgery within 6 weeks after the end of the neo-adjuvant treatment. The phase II part of the study will start if all endpoints are met and after approval of an independent data monitoring committee (IDMC) that will review safety data of the 6 patients included in the safety run-in phase. The dosages of the IMPs will be identical in the safety run-in and the phase II randomized trial.

#### Primary objective and endpoint

The primary objective of the study is to demonstrate an improved tumour response rate after neoadjuvant therapy at surgery with minimal or no residual disease in the primary tumour and in the lymph nodes in arms 2 or 3 versus arm 1. Residual disease will be measured using the residual cancer burden (RCB) index on the surgical specimen. RCB is calculated as a continuous index combining pathologic measurements of the primary tumour (size and cellularity) and lymph node metastases (number and size) [12]. The primary endpoint will be the rate of RCB 0 or 1 at time of surgery. RCB 0 is defined as pCR and RCB 1 is defined as minimal residual disease.

#### Secondary objectives and endpoints

Secondary objectives are to 1) evaluate the response of the primary tumour irrespective of the response to the pathological lymph nodes; 2) evaluate the response of the pathological lymph nodes irrespective of the response to the primary tumour. These two objectives are of special interest to differentiate the local impact of radiation therapy, which is exclusively directed to the primary cancer and to not to the lymph nodes. Other objectives are to: 3) evaluate the feasibility of performing breast-conserving surgeries in arms 2 and 3 versus arm 1; 4) evaluate invasive disease-free survival (iDFS) in arms 2 and 3 versus arm 1 at 3 years after surgery; 5) evaluate the severity and duration of adverse events in arms 2 and 3 versus arm 1; 6) evaluate the cosmetic changes to the breast in arms 2 and 3 versus arm 1.

#### Translational objectives and endpoints

The main translational objectives are to 1) evaluate TILs levels in arms 2 and 3 versus arm 1 at different timepoints during the study and correlate these findings to RCB-category [13] and 2) identify potential predictive immune biomarkers by performing a widespread histological and molecular profiling of the pre-, on-, and post-study treatment peripheral blood and tumour tissues, by using immuno- histochemistry and other histological methods such as immunofluorescence, flow cytometry, chromatography and next generation sequencing. Biomarkers include TILs, PD-L1, CD73 and BMI [14] and 3) evaluate the prognostic importance of germinal centers in lymph nodes after treatment and relationship to TILs.

### Study population

#### Inclusion criteria

##### Main inclusion criteria

- Histological diagnosis of invasive breast adenocarcinoma that is **ER-positive** and **HER2-negative** as per the updated American Society of Clinical Oncology (ASCO) - College of American Pathologists (CAP) guidelines [15] according to local testing and using validated assays in ISO15189-accredited laboratories.
  - ER-positive is defined as having an immunohistochemistry (IHC) of 10% or more and/or and Allred score of 3 or more
  - HER2-negative is defined as having an IHC of 0 or 1+ without in-situ hybridization (ISH) testing OR IHC 2+ and ISH non-amplified with ratio less than 2.0 and if reported, average HER2 copy number < 4 signals/cells OR ISH non-amplified with ratio less than 2.0 and if reported, average HER2 copy number < 4 signals/cells [without IHC]; note: a IHC of 3+ is always considered HER2 positive, independently of the ISH result.
- **Luminal B** breast tumour, ascertained by a Proliferation Index Ki67 ≥ 15% or histological grade 3.
- Confirmed **MammaPrint genomic high risk**.
- **cN0** or c**N1** according to TNM classification
- Absence of distant metastasis (**cM0**)
- **Multifocal, multicentric** unilateral or **bilateral** breast adenocarcinomas are allowed provided that all foci are ER+/HER2- according to local testing and all foci are able to receive pre-operative radiation therapy treatment within the defined dosimetric constraints.

*The complete list of exclusion criteria can be found in the supplementary materials.*

##### Exclusion criteria

*Main exclusion criteria:*

- TNM stage **cT4** BC, including inflammatory BC
- Presence of any **distant metastasis**

*The complete list of exclusion criteria can be found in the supplementary materials.*

### Treatments and interventions

#### Treatments

The chemotherapy backbone of the trial consists of 12 administrations of intravenous (IV) weekly paclitaxel (80 mg/m^2^) followed by 4 administrations of doxorubicin (60 mg/m^2^) plus cyclophosphamide (600 mg/m^2^ IV) in a dose dense scheme (ddAC), i.e. every 2 weeks (q2w) (Table 1). Durvalumab (anti PD-L1) is given q4w 1500 mg IV. Oleclumab (anti-CD-73; MEDI9447) is given q2w for 5 administrations, then q4w during 2 administrations (3000 mg IV). A detailed overview can be found in the study scheme (Fig. 1).

**Table 1:** Pre-operative trial treatment schedule and dose

Drug or treatment	Dose and route	Frequency	Total number of treatments	Treatment period
Paclitaxel	80 mg/m^2^ IV	Q1w	12	Week 1 until week 12
Doxorubicin	60 mg/m^2^ IV	Q2w	4	Week 13 until week 19
Cyclophosphamide	600 mg/m^2^ IV	Q2w	4	Week 13 until week 19
Radiation therapy to the primary breast cancer	8 Gy	Daily	3	Just before week 5
Durvalumab	1500 mg IV	Q4w	5	Week 1 until week 17
Oleclumab	3000 mg IV	Q2W for 5 administrations, then Q4W during 2 administrations	7	Week 1 until week 17

Pre-operative radiation therapy to the primary tumour will be given at a dose of 24 Gy in 3 fractions using an SBRT technique with the 3rd fraction given on the same day as the 5th paclitaxel, 2nd durvalumab and 3rd oleclumab administration (Fig. 1). Pathological lymph nodes will not be irradiated. Special care will be taken to reduce the dose to the axillary lymph nodes, the heart, the lungs and the skin. To allow for optimal image guidance during SBRT treatment, at least 3 MRI-compatible markers must be placed in the primary tumour during the screening period. Image guidance during treatment will be performed using daily CBCT (cone beam computed tomography) and markers-based matching. MRI in supine position, preferably in radiation therapy treatment position, is recommended to facilitate contouring.

Surgery should be planned 2–6 weeks after completion of ddAC and will consist of breast conserving surgery or mastectomy and a sentinel node procedure and/or an axillary lymph node dissection.

#### Interventions

The study design (Fig. 1) charts the timing of all scheduled blood samplings, biopsies and tumour imaging procedures. A new biopsy of the primary tumour is planned 1 week after ending the radiation therapy (i.e. week 6) to evaluate tumour and tumour microenvironment changes induced by the first 5 weeks of paclitaxel, durvalumab, oleclumab and radiation therapy. An MRI is planned 12 weeks after the start of study treatment, before switching the chemotherapy to ddAC. Blood samples for translational research are scheduled throughout the pre-operative phase.

#### Treatments after surgery

Adjuvant endocrine therapy is given according to standard of care (SOC). Post-operative radiation therapy will be administered according to SOC, with the only exception that no boost to the tumour bed is allowed, because the pre-operative radiation therapy to the primary tumour is to be considered as an anticipated boost.

### Statistical analysis

#### Sample size

Six patients will be included in the safety run-in. In the phase II part, patients will be randomized in a 1:1:1 ratio between 3 arms and the primary endpoint that will be measured on those patients is the binary achievement of RCB 0–1 versus RCB 2–3. The trial is designed to compare arm 2 to arm 1 as well as arm 3 to arm 1. The trial is not powered to compare arms 2 and 3 and no specific hypothesis is formulated for that comparison. It is expected that arm 1 will achieve an RCB 0–1 rate of 15% and the experimental treatment arms will be considered of interest for further investigation if this rate can be increased to 45%. This estimation for arms 2 and 3 are based on the first results of the I-SPY 2 trial (2017): the addition of immune checkpoint blockade (ICB) to standard NACT increased pCR from 13.6 to 34.2% [16]. Taking into account the addition of radiation therapy and the use of RCB 0–1 as endpoint, we hypothesize an increase in RCB 0–1 rates from 15 to 45% in either arm 2 or 3. There will be then two hypotheses testing, both aiming to reject the null hypothesis of equality of the RCB 0–1 rates between the control and in the experimental arms. A two-sided alpha level of 2.5% will be used and a power of 80% has been targeted in case the true RCB rate in either arm 2 or arm 3 is at least 45%. The alpha level of 2.5% was chosen to adjust for multiplicity. No continuity correction was used, and a pooled estimate of variance was chosen to estimate requested sample size. Sample size calculations are summarized in Table 2. With those assumptions, 44 evaluable patients per arm are needed, i.e. a total of 132 patients. In order to take into account a 5% non-evaluability rate and a 5% non-eligibility rate, this sample size will be increased to 147 patients to be randomized. Assuming 20% screening failures (taking into account MammaPrint patients that are screened as MammaPrint genomic low risk), 184 patients will have to be screened.

**Table 2:** Statistical sample size calculation

**Safety run-in**	
Numbers of subjects needed	6 subjects
**Phase II trial**	
Primary endpoint achievement RCB 0/1 vs. RCB 2/3	Standard arm: 15%
	Experimental arms: 45%
	Hypothesis: increase in RCB 0/1 rates from 15 to 45% in either arm 2 or 3.
Alpha level (two-sided)	2.5%
Power	80%
Number of subjects needed	
1:1:1 randomization	44 subjects per arm, total of **132** subjects
Increase for:	
• 5% ineligible after randomisation	Add 15 subjects
• 5% unevaluable subjects	147 randomised subjects
Increase for 20% ineligible after screening (MammaPrint genomic low risk).	Add 37 subjects.
**Total for phase 2 trial**	184 subjects screened
**Total evaluable subjects (safety run-in + phase II)**	**136 subjects**

#### Data analysis

Primary endpoint analysis will be carried out on the eligible and randomized patients who have been operated and in whom the residual cancer burden was measured. The observed proportions will be compared by a chi square test without continuity correction at an alpha two-sided level of 2.5%. Confidence intervals for the difference between proportions will be provided at the usual 95% level. The comparison of RCB 0–1 rates between arm 2 and arm 1 as well as arm 3 and arm 1 will be performed. As exploratory analysis, a confidence interval for the difference between proportions in arm 2 and arm 3 will be provided.

## Discussion

Approximately 40% of patients with early-stage luminal B BC who receive NACT experience recurrent disease within 5 years [5], therefore new treatment strategies to decrease recurrence rates are greatly needed. In recent years, an emerging treatment strategy is the use of ICB. Early trials of single-agent ICB in BC show evidence of a modest response, less than in some other cancers due to the low baseline immunogenicity of most BCs [17]. Recent advances in the field of immunogenomics identified different immune subtypes that are hypothesized to define how immune response patterns impact prognosis [18]. In BC, the immune response of hormone receptor positive (luminal) BC is lower compared to the TNBC and HER2+ subtypes, assumedly because luminal BC is generally less inflamed, characterized by a lower presence of TILs and lower PD-L1 expression [19]. In TNBC and HER2+ BC, increased TIL concentration is correlated with response to chemotherapy and immunotherapy, and is associated with increased survival, whereas in luminal BC the prognostic and predictive value of increased TIL concentration is not fully established [7].

An attractive strategy to increase the benefit of immunotherapy in luminal B BC seems to be to prime the immune response using a combination strategy with chemotherapy, a doublet immunotherapy, radiation therapy or other agents. The I-SPY2 trial investigated the addition of pembrolizumab (anti PD-1) to a standard NACT backbone in TNBC and luminal B BCs [20]. The addition of pembrolizumab increased pCR rates from 13 to 30% in luminal B BC and from 22 to 60% in TNBC. These results indicate that non-inflamed” tumours might benefit from immunotherapy when combined with other treatments, such as chemotherapy. In TNBC, the phase III trial KEYNOTE 522 demonstrated superiority of the pembrolizumab-NACT combination with 64.8% pCR and 51.2% pCR in the NACT-only group [21], whereas for luminal B BC, KEYNOTE 756 is ongoing.

An active domain of research is the identification of non-chemotherapeutic agents able to prime the immune response and to further enhance the conversion to more inflamed tumours with the hope of increasing response rates to immunotherapy. In the Neo-CheckRay trial, radiation therapy to the primary tumour and the use of an anti-CD-73 are investigated as potential strategies to increase the response rate following immunotherapy-chemotherapy combinations.

In recent years, a large amount of clinical trials and animal studies have described the synergistic effects on local and distant tumour control of combining radiation therapy with immunotherapy [22–26]. In BC models, radiation has been shown to induce T cell priming via antigenic release and by activation of the innate immunity [22, 27, 28]. Pivotal pre-clinical work by Vanpouille-Box et al. showed that a fractionation schedule of three fractions of 8 Gy given daily induces a better activation of the cGAS-STING pathway in comparison to treatments with a higher dose per fraction [29]. Despite the fact that the 3 × 8 Gy schedule has been used in several clinical trials to evaluate its efficacy and toxicity in combination with immunotherapy [30–32], this fractionation schedule remains yet to be validated. While an excessively high radiation fraction dose might suppress the immune response or increase toxicity, an insufficient dose might not induce an immune response at all. Apart from dose/fractionation parameters, also the sequencing of radiation therapy when combined with ICB remains largely unknown, further highlighting the importance of the need for more clinical trials. A challenge of delivering pre-operative radiation therapy in combination with immunotherapy is the concern of post-operative toxicities, with potential impacts on breast cosmesis, lymphedema, fibrosis and surgical outcomes, especially in case of breast reconstruction. The impact on these parameters can be estimated from other pre-operative trials combing radiation therapy with chemotherapy [33], but it remains unknown how the addition of immunotherapy will modulate these effects. For this, breast cosmesis will be monitored during and after the Neo-Checkray trial, using standardised breast photography and clinical examinations.

Oleclumab is a human immunoglobulin monoclonal antibody (mAb) that selectively binds to and inhibits the ectonucleotidase activity of CD73. CD73 converts adenosine monophosphate (AMP) into adenosine. Adenosine presents anti-inflammatory and immunosuppressive effects, mediated by the adenosine receptors expressed on immune cells [34, 35]. CD73 therefore serves as an immune checkpoint, with the clinical association between elevated CD73 expression and poor prognosis being well documented in several tumour types [36–38]. The therapeutic potential of blocking adenosine pathways with monoclonal antibodies targeting is currently being tested in phase II trials [39].

Radiation therapy has pre-clinically been shown to act synergistically with anti-CD-73 through the prevention of adenosine-mediated immunosuppression [40]. Recent research suggests that CD73 may be a radiation-induced checkpoint, and that CD73 blockade in combination with radiation therapy and ICB might improve patient response to therapy [41]. Pre-clinical data show that the combination of CD73 inhibition and radiation therapy has the following effect: 1) enhancement of the radiation-induced activation of the antitumour immune response, 2) restriction of the immunosuppressive action of CD39/CD73 on circulating immune cells and 3) attenuation of adverse late effects of radiation therapy through inhibition of fibrosis [41–43].

The Neo-CheckRay trial was designed to harness these potential synergies, by combining durvalumab with oleclumab and radiation therapy to the primary BC in addition to a standard chemotherapy backbone. Parts of this combination have been tested for safety, such as the combination of radiation therapy and docetaxel [33], radiation therapy with PD-L1 blockade [32], and the combination of oleclumab, durvalumab and paclitaxel [44]. The combination of all 4 treatment agents will be tested in a safety run-in phase of the Neo-CheckRay trial before proceeding to the randomized phase II trial.

Given the choice of RCB 0–1 as primary endpoint of the current trial, it was decided to deliver radiation therapy to the three treatment arms to avoid an imbalance in local treatment between the study arms. Indeed, radiation therapy might increase the local response without necessarily reflecting an impact on the systemic response and is therefore more difficult to discriminate as being an independent surrogate of long-term outcome. Moreover, our study design allows singling out the effect of durvalumab and oleclumab without the results being disbalanced by radiation therapy. The drawback of this choice is that arm 1 is not a commonly accepted standard of care, which would have been chemotherapy alone, excluding the individual impact of pre-operative SBRT.

A similar trial could have been designed with the systemic treatments in the adjuvant setting or in the metastatic setting. The neo-adjuvant setting, however, with a primary tumour in place, presents unique advantages for priming the anti-tumour immune response and potential eradication of micrometastatic disease [45]. Pre-clinical models of BC support the superiority of neo-adjuvant immunotherapy compared to adjuvant immunotherapy [46]. With the intact primary cancer still in place, radiation therapy and ICB might help to convert the tumour into an in-situ, individualized vaccine; hypothetically preventing patients against metastasis [47]. In addition, neo-adjuvant treatment followed by complete surgical resection of the tumour allows a rapid and thorough assessment of response with the possibility of performing a wide range of translational research on the surgical specimen.

The Neo-Checkray trial targets a subselection of luminal B cancers by strictly including patients with a MammaPrint test showing a genomic high risk of relapse. Patients with luminal B BC and a genomic high risk of relapse derive a greater benefit from chemotherapy than patients with a genomic low risk result [48]. The use of this test ensures that patients included in the trial derive benefit of chemotherapy. Unlike with chemotherapy, biomarkers to select patients for immunotherapy have not yet been identified for BC patients. Hence, an important part of the translational research objectives of the Neo-CheckRay study is to evaluate the predictive value of potential biomarkers, such as PD-L1 and TILs, and to identify other biomarkers, both in situ as well as in the systemic circulation [49], that might help to better tailor therapy in this setting.

Few other trials are investigating the role of pre-operative radiation therapy in combination with immunotherapy in BC. A phase I clinical trial (NCT03366844 - recruiting) is evaluating the safety of 3 × 8 Gy radiation therapy to the primary BC in combination with pembrolizumab for TNBC and ER + HER2- BC, without the use of chemotherapy [50]. Another pre-operative design in luminal BC is the CBCV trial (NCT03804944 – not yet recruiting), a 4 arms study consisting of 1) radiation therapy 3 × 8 Gy (RT) + hormonal treatment (HT); 2) RT/HT + pembrolizumab; 3) RT/HT + CDX-301 (anti-CD135); 4) RT/HT + pembrolizumab + CDX-301 [51]. In TNBC, the PANDoRA trial (NCT03872505 – not yet recruiting) will evaluate the addition of radiation therapy (3x8Gy) to non-anthracycline chemotherapy and durvalumab in TNBC [52]. To our knowledge, the Neo-CheckRay trial is the first trial to examine the effect of the pre-operative addition of radiation therapy to the combination of immunotherapy with standard NACT in early luminal B BC.

## Data Availability

Data sharing is not applicable to this article as no datasets were generated or analysed yet during the current study.

## Abbreviations

AMP: Adenosine monophosphate
BC: Breast cancer
CBCT: Cone beam computed tomography
ddAC: dose-dense doxorubicin-cyclophosphamide
ER: Estrogen receptor
HT: Hormonal treatment
ICB: Immune checkpoint blockade
iDFS: invasive disease-free survival
IDMC: Independent data monitoring committee
IHC: Immunohistochemistry
IMP: Investigational medicinal product
ISH: In-situ hybridization
IV: Intravenous
mAb: monoclonal antibody
NACT: Neo-adjuvant chemotherapy
pCR: pathological complete response
PD-L1: Programmed cell death receptor ligand 1
RCB: Residual cancer burden
SBRT: Stereotactic body radiation therapy
SOC: Standard of care
TIL: Tumor infiltrating lymphocyte
TNBC: Triple negative breast cancer
